# Penetrating Chest Trauma With Right Atrial Rupture: A Case Report

**DOI:** 10.7759/cureus.75171

**Published:** 2024-12-05

**Authors:** Menawar Dajenah, Faisal Ahmed, Anessa Thabet, Zaid M Dajenah

**Affiliations:** 1 Department of General Surgery, School of Medicine, Faculty of Medicine and Health Science, Ibb University, Ibb, YEM; 2 Department of Urology, School of Medicine, Faculty of Medicine and Health Science, Ibb University, Ibb, YEM; 3 Department of Gynecology, School of Medicine, Faculty of Medicine and Health Science, Ibb University, Ibb, YEM; 4 Student Research Unit, School of Medicine, 21 September University, Sana'a, YEM

**Keywords:** cardiac tamponade, case report, hemopericardium, penetrating chest trauma, right atrial rupture, thoracotomy

## Abstract

Penetrating thoracic injuries, especially those affecting cardiac structures, are rare but can be life-threatening, requiring urgent medical care. Right atrium injuries pose significant risks, including rapid blood loss, cardiac tamponade, hemodynamic instability, and, subsequently, potential death. We report the case of a 24-year-old male patient with stab wounds leading to a right-sided chest penetration three hours prior to presentation. The patient exhibited hypotension, tachycardia, and mild respiratory distress upon arrival. The patient had a 1.5x3 cm penetrating injury in the right fourth intercostal space and another 1x3 cm incision in the fifth intercostal region. A right chest tube was inserted, draining 500 milliliters of hemothorax, and continued to increase without improvement in the patient's condition. The patient was quickly taken to the operating room after initial resuscitation with fluids and blood transfusion, where the abdominal diagnostic laparoscopy was negative, but a thoracotomy revealed hemopericardium and a 2x3 cm right atrial rupture. A tangential Satinsky clamp and a double-layered 5-0 double-arm polypropylene suture were utilized to achieve hemostasis during repair. The patient was extubated successfully on the first postoperative day and discharged in stable condition on the ninth day. This case underscores the importance of prompt diagnosis and surgical management in cases of penetrating cardiac trauma. Even in resource-limited settings, managing right atrial rupture can be effectively achieved through rapid resuscitation, proper imaging, and skilled surgical techniques.

## Introduction

Penetrating thoracic injuries, especially those affecting cardiac structures, pose significant clinical challenges due to high mortality rates [1]. Cardiac injuries involving the right atrium have a dire prognosis due to risks of rapid bleeding, cardiac tamponade, and hemodynamic instability. Timely identification and surgical intervention are crucial for improving survival rates, as even slight treatment delays can have severe outcomes [2,3].

Although penetrating trauma is less common than blunt trauma as it accounts for only 0.1% of most trauma admissions, it results in higher fatality rates due to proximity to critical cardiac and vascular structures [4]. Penetrating cardiac injuries, often from stabbings, are typically linked to severe assaults and accidents [5]. The anatomical vulnerability of the right atrium may result in life-threatening bleeding. However, timely intervention remains problematic in resource-limited settings, with positive outcomes dependent on early diagnosis and skilled surgical personnel [1,5,6].

Penetrating injuries to the anterior chest wall can result in fatal damage to various thoracic structures, yet reports on these injuries and their management are scarce [7]. Furthermore, documenting such cases is essential for enhancing surgical techniques, particularly in low-resource environments [1,7]. This report discusses a case of penetrating chest trauma with a right atrium injury from a stab wound, effectively managed through rapid resuscitation and decisive surgical intervention.

## Case presentation

**Figure 1:**
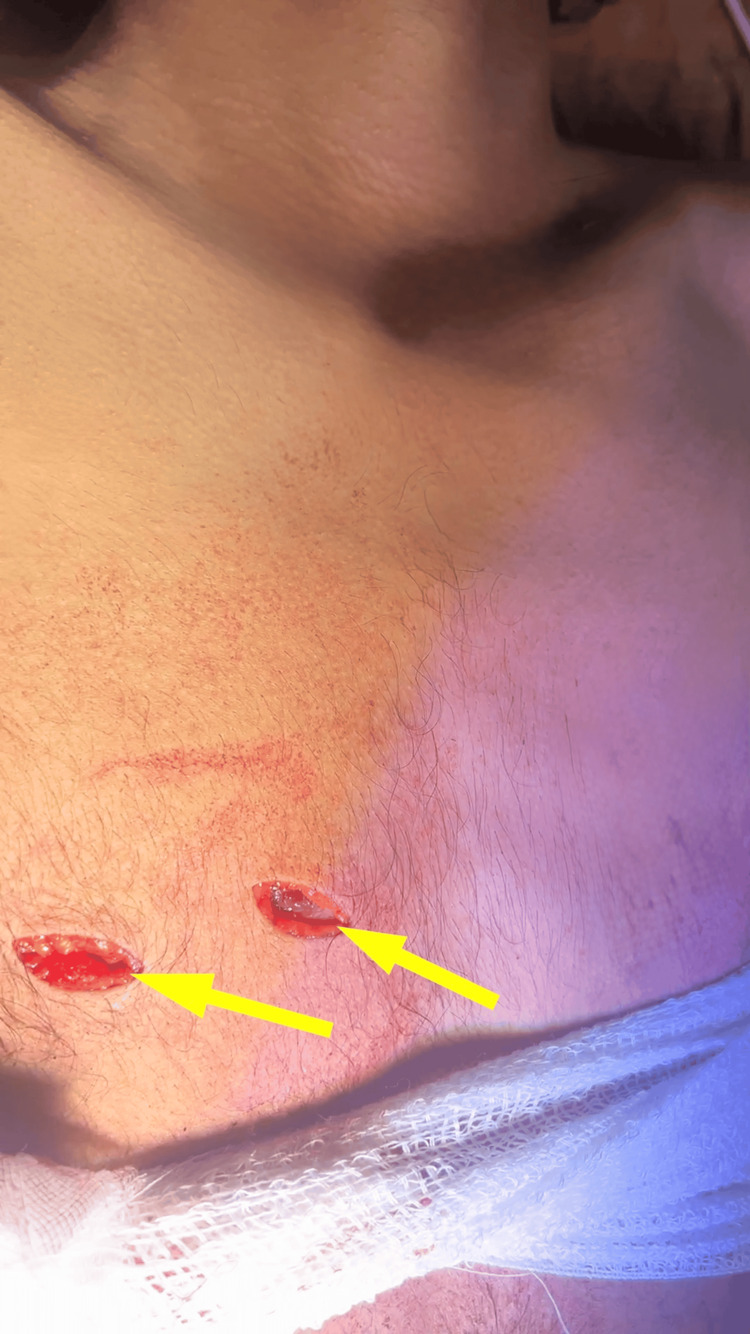
Chest wall injuries (yellow arrows).

A 24-year-old male patient presented to the emergency department with a documented history of stab wounds located in the anterior precordial region, which penetrated the chest on the right side, approximately three hours post injury. Upon examination, the patient demonstrated a penetrating injury measuring 1.5x3 cm situated in the right fourth intercostal space within the parasternal region with an additional penetrating wound measuring 1x3 cm located at the fifth intercostal space along the midclavicular line (Figure 1).

The patient was assessed as semi-conscious and reported experiencing intense pain on the right side of the chest and respiratory distress while exhibiting signs of hemodynamic instability and an absence of air entry in the right thoracic cavity. The initial evaluation indicated a blood pressure of approximately 80/50 mmHg, a pulse rate of 150 beats per minute, a respiratory rate of 30 breaths per minute, jugular vein distention, and muffled heart sounds. Oxygen saturation was recorded at 90% while the patient received 2 liters of supplemental oxygen. After initial resuscitation efforts, the blood pressure improved to 90/50 mmHg, although the patient continued to experience respiratory difficulties and persistent hemodynamic instability. Immediately, a chest tube was inserted to address the right-sided hemothorax, evacuating 500 ml of blood upon insertion and continued to increase. Due to the patient's unstable condition, no radiologic investigations were undertaken.

Within 15 minutes of hospital arrival, the patient was promptly transferred to the operating room after initial resuscitation with fluids and blood transfusion. A central venous catheter was inserted, and a broad-spectrum antibiotic was administered following induction. Firstly, a diagnostic laparoscopy was made to investigate the abdomen. The diaphragm, liver, spleen, and other abdominal organs were all examined and found intact. To treat the probable hemothorax and cardiac tamponade, the patient was positioned supine with the right side of the chest slightly elevated. Upon examination of the penetrating chest wounds, active bleeding from multiple intercostal vessels was identified and promptly addressed through suturing. The thoracic cavity was explored to palpate the diaphragm and assess for potential injuries. A defect in the pericardium was detected, necessitating lateral extension of the wound to evaluate the status of the underlying lungs and pericardium.

**Figure 2:**
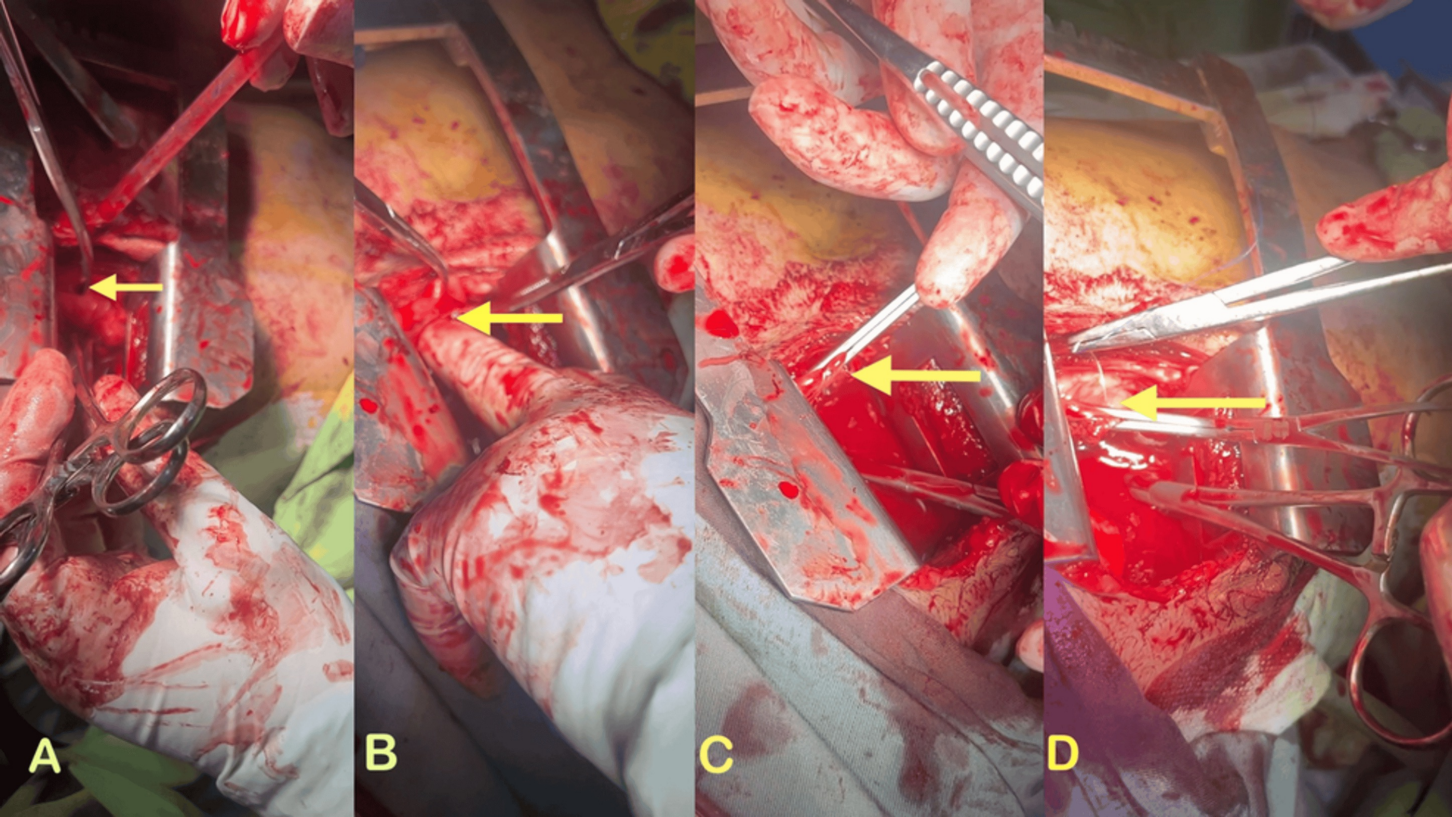
Intraoperative photos showing (A) Pericardial breach (yellow arrow); (B) Pericardial laceration and rupture of the right atrial wall were observed and briefly stabilized using the index finger (yellow arrow); (C) Right atrial wall clamped with vascular clamp (yellow arrow); (D) Repaired using a 5-0 double-arm polypropylene suture in double layers until hemostasis was achieved (yellow arrow)

During the surgical intervention, a pericardial tear measuring approximately 1.5×1.5 cm was observed, accompanied by clot formation and hemopericardium (Figure 2A). Following the evacuation of the clot and blood from the pericardial space, a laceration and rupture of the right atrial wall were observed and briefly stabilized using the index finger during the thoracotomy (Figure 2B). A tangential Satinsky clamp was applied to the lacerated area of the right atrial wall (Figure 2C). The heart rate decreased from 160 to 115 beats per minute, and blood pressure improved to 90/60 mmHg. The clamped right atrial wall was repaired utilizing a 5-0 double-arm polypropylene suture in a double-layer technique until hemostasis was successfully achieved (Figure 2D). The thoracic cavity was subsequently re-examined, the lung injury was addressed, and comprehensive hemostasis was ensured. The thoracic cavity was then rinsed with normal saline and closed in layers following the insertion of two chest drains-one positioned in the pericardium and the other within the chest cavity. The total blood transfusion during the operation was three packed cells, the total operative time was 120 minutes, and the total bleeding was 2300 cc.

**Figure 3:**
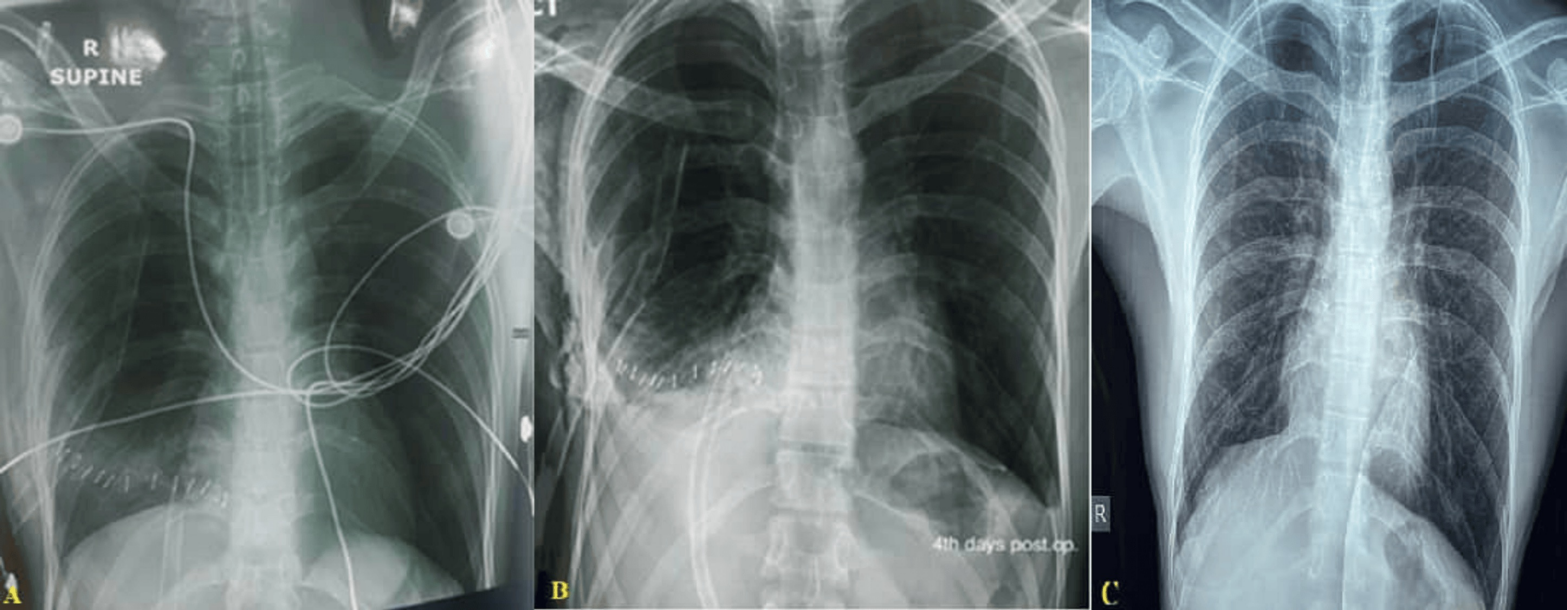
Postoperative radiologic X-ray showed no abnormalities (A) on the first postoperative day, (B) the fourth postoperative day, and (C) at the three-month follow-up

The patient was transferred to the intensive care unit for further evaluation, monitoring, and management. He was extubated on the first postoperative day and transferred to the general surgery ward on the third postoperative day. Chest X-rays showed no abnormalities (Figure 3A). The chest tube was removed on the seventh day, and the patient was discharged on the ninth postoperative day (Figure 3B). The patient was doing well after three and seven months of follow-up, and chest X-ray and echocardiography investigations revealed no abnormalities (Figure 3C).

## Discussion

Penetrating thoracic trauma resulting in right atrial rupture constitutes a rare yet critical injury that demands immediate assessment and surgical intervention [1,4]. Due to its specific anatomical positioning, the heart is particularly vulnerable to penetrating injuries, with right atrial rupture posing a considerable mortality risk if not addressed expeditiously [7,8]. The case reported by Valdés-Dupeyrón et al. exemplifies the imperative need for rapid resuscitation and suitable surgical management to secure patient survival, as was seen in our case [8]. In the presented case, the patient sustained a penetrating stab wound to the right thoracic region, leading to hemopericardium and rupture of the right atrial wall. The successful application of the vascular clamp was followed by meticulous suturing of the atrial rupture, resulting in effective hemostatic control and stabilizing the patient's clinical status. Deploying double-arm polypropylene sutures in a layered fashion was instrumental in achieving hemostasis, a technique well-established in cardiac surgical practice for managing such catastrophic injuries [1,7].

Historically, cardiac injuries have been associated with dire prognoses and were deemed intractable; presently, approximately 90% of individuals succumb before arrival at the emergency department [9]. Numerous researchers have identified correlations between mortality and the hemodynamic status of the patient upon admission, the type of weapon utilized, surgical findings, and the intricacy of the repair process [10]. In our clinical scenario, the medical emergency team's intervention was indispensable for preserving these patients' lives. Cardiac injury should be anticipated in any patient presenting with penetrating injuries to the thoracic region, particularly on the anterior aspect of the thorax, predominantly on the left side, as well as in the upper abdomen and neck [8,11].

Numerous researchers acknowledge that the survival rate following a penetrating cardiac injury is likely contingent upon the timeliness of medical intervention [9]. Stranch et al. discovered a statistically significant correlation between the delay in hospital arrival, the clinical status upon admission, the mechanism of injury, and the implementation of aggressive surgical treatment with patient survival rates [12]. The time between injury and operation for penetrating heart damage must be brief. Isaza et al. observed that the average was 60 minutes [7]. Survival after a penetrating cardiac injury appears to be less likely in a patient five hours after the trauma. However, penetrating wounds in the anatomical area known as the "cardiac box" should raise the most concerns for penetrating heart damage [4]. Focused and coordinated surveys, as well as resuscitation, are beneficial to any patient with penetrating thoracic trauma. In the present case, the time to surgical intervention was about three hours. In another report, Gucho et al. reported a delayed presentation of penetrating cardiac injury for five hours in a 21-year-old patient with a stab wound on his right chest's fourth intercostal space [4].

Penetrating traumas to any of the cardiac chambers may precipitate acute cardiac tamponade and subsequent mortality rapidly. Hemorrhagic flow resulting from a lacerated pericardial injury will disseminate into the hemithorax, ultimately culminating in a fatality. Consequently, the pericardium serves a critical role in averting lethal exsanguination and enables patients to endure sufficiently long to attain medical intervention at a trauma center. Patients may exhibit various degrees of hemodynamic instability as a consequence of pericardial tamponade [9]. Notably, the classical clinical manifestation of Beck’s triad (characterized by muffled heart sounds, jugular venous distension, and hypotension) or Kussmaul’s sign (indicative of jugular venous distension upon inspiration) is observed in merely 10% of patients presenting with pericardial tamponade [9].

Penetrating trauma can result in a pneumothorax or haemothorax with significant blood loss. Some individuals see fast deterioration following chest injuries. However, with correct care, they can quickly improve. In patients with penetrating injuries, surgical operations are typically suggested, although diagnostic examinations are less necessary than in blunt trauma [13]. Various surgical strategies exist for addressing penetrating cardiac trauma, including left anterior thoracotomy, right anterior thoracotomy, pericardial window, and median sternotomy. The latter approach is prevalent owing to its extensive exposure, albeit it is not as expeditious as other techniques [14,15].

In our case, we opted for an abdominal laparoscopic diagnostic procedure without prior radiological assessment due to the patient's declining condition. Consequently, we prioritized abdominal examination over thoracic assessment, believing the abdominal and hepatic injuries were of greater severity. Upon negative findings, the surgical strategy shifted to thoracotomy and pericardial exploration, resulting in the identification and repair of the atrial injury; a comparable case with certain deviations was documented by Alfraidy et al. [1]. In a separate report by Valdés-Dupeyrón et al., the authors conducted a left thoracotomy combined with a transverse sternotomy in one patient, where a conventional sternotomy would have delayed vascular control [8].

Despite the rarity of penetrating cardiac injuries in low-resource settings, favorable results are possible with appropriate resuscitation and surgical capabilities [4,6]. The present case contributes to the existing literature on addressing similar injuries in economically weak healthcare systems with inadequate resources.

## Conclusions

Penetrating thoracic trauma that affects the right atrium represents a rare yet potentially life-threatening condition that requires immediate assessment and intervention. This case exemplifies the critical nature of rapid diagnosis, proficient resuscitation, and thorough surgical repair in managing such injuries. The effective utilization of a tangential Satinsky clamp coupled with double-layer polypropylene sutures to control bleeding demonstrates that survival can be achieved even in severe cases of cardiac injury. Furthermore, this case further emphasizes the need to follow standard principles of resuscitation and guidelines for the management of thoracic trauma in places with a high incidence of penetrating chest trauma. Applying clinical diligence and the use of imaging like CT trauma series after stabilizing these patients could avoid potentially morbid negative explorations. Even in resource-poor settings, the use of emergency room/bedside ultrasound or diagnostic peritoneal lavage for abdominal injuries could avoid such aggressive exploratory attempts.
